# The genetic overlap between mood disorders and cardiometabolic diseases: a systematic review of genome wide and candidate gene studies

**DOI:** 10.1038/tp.2016.261

**Published:** 2017-01-24

**Authors:** A T Amare, K O Schubert, M Klingler-Hoffmann, S Cohen-Woods, B T Baune

**Affiliations:** 1 Discipline of Psychiatry, School of Medicine, The University of Adelaide, Adelaide, SA, Australia; 2 Northern Adelaide Local Health Network, Mental Health Services, Adelaide, SA, Australia; 3 Adelaide Proteomics Centre, School of Biological Sciences, The University of Adelaide, Adelaide, SA, Australia; 4 School of Psychology, Faculty of Social and Behavioural Sciences, Flinders University, Adelaide, SA, Australia

## Abstract

Meta-analyses of genome-wide association studies (meta-GWASs) and candidate gene studies have identified genetic variants associated with cardiovascular diseases, metabolic diseases and mood disorders. Although previous efforts were successful for individual disease conditions (single disease), limited information exists on shared genetic risk between these disorders. This article presents a detailed review and analysis of cardiometabolic diseases risk (CMD-R) genes that are also associated with mood disorders. First, we reviewed meta-GWASs published until January 2016, for the diseases ‘type 2 diabetes, coronary artery disease, hypertension’ and/or for the risk factors ‘blood pressure, obesity, plasma lipid levels, insulin and glucose related traits’. We then searched the literature for published associations of these CMD-R genes with mood disorders. We considered studies that reported a significant association of at least one of the CMD-R genes and ‘depression’ or ‘depressive disorder’ or ‘depressive symptoms’ or ‘bipolar disorder’ or ‘lithium treatment response in bipolar disorder’, or ‘serotonin reuptake inhibitors treatment response in major depression’. Our review revealed 24 potential pleiotropic genes that are likely to be shared between mood disorders and CMD-Rs. These genes include *MTHFR*, *CACNA1D*, *CACNB2*, *GNAS*, *ADRB1*, *NCAN*, *REST*, *FTO*, *POMC*, *BDNF*, *CREB*, *ITIH4*, *LEP*, *GSK3B*, *SLC18A1*, *TLR4*, *PPP1R1B*, *APOE*, *CRY2*, *HTR1A*, *ADRA2A*, *TCF7L2*, *MTNR1B* and *IGF1*. A pathway analysis of these genes revealed significant pathways: *corticotrophin-releasing hormone signaling*, *AMPK signaling*, *cAMP-mediated or G-protein coupled receptor signaling*, *axonal guidance signaling*, *serotonin or dopamine receptors signaling*, d*opamine-DARPP32 feedback in cAMP signaling*, *circadian rhythm signaling* and *leptin signaling*. Our review provides insights into the shared biological mechanisms of mood disorders and cardiometabolic diseases.

## Introduction

Major depressive disorder (MDD), bipolar disorder (BPD), coronary artery diseases, type 2 diabetes and hypertension are amongst the major causes of disability, morbidity and mortality worldwide.^1, 2^ Although each of these conditions independently represent a major burden facing the health-care systems,^1, 2, 3^ their co-occurrence (co-morbidity) aggravates the situation and represents a challenge in psychosomatic medicine.^4^ Epidemiologically, MDD and BPD are bi-directionally associated with cardiometabolic diseases.^5, 6^ A similar pattern of association has been shown in the relationship between the pharmacological treatment of mood disorders and cardiometabolic diseases. For instance, the use of antidepressants and mood stabilizers is associated with an increased risk of cardiometabolic abnormalities^7^ and cardiac medications might increase the risk of mood disorders.^8^ One explanation for these relationships could be the presence of pleiotropic (common) genes and shared biological pathways that function as a hub to link the disorders. Potential common biological mechanisms underlying mood disorders and cardiometabolic disease comorbidity have been proposed, including altered circadian rhythms,^9^ abnormal hypothalamic–pituitary–adrenal axis (HPA axis) function,^10^ imbalanced neurotransmitters^11^ and inflammation.^6^ However, the molecular drivers of these commonly affected mechanisms remain poorly understood.

## The genetics of mood disorders and cardiometabolic diseases

**Figure 1:**
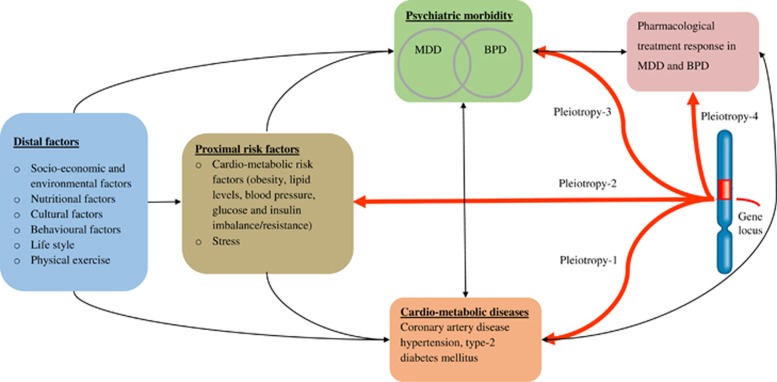
Schematic model for the potential pleiotropic effects of a shared gene locus that is associated with mood disorders and cardiometabolic diseases.^5, 6, 26, 70, 71, 75^ The distal and proximal factors are obtained from the literature, and the World Health Organization (WHO) often uses the classification. Distal factors refer to those factors that require an intermediate factor to cause diseases, while proximal factors can directly cause diseases. The red bold lines represent the pleiotropic effect of a genetic locus on cardiometabolic diseases and associated risk factors, psychiatric morbidity, i.e.mood disorders and pharmacological treatment response in MDD and BPD. The bi-directional arrows indicate bidirectional epidemiological relationships between the cardiometabolic diseases and mood disorders. BPD, bipolar disorder; MDD, major depressive disorder.

Major depression, bipolar disorder and cardiometabolic diseases are highly heritable and they are caused by a combination of genetic and environmental factors. Genetic factors contribute to 31-42% in MDD,^12^ 59–85% in BPD,^13, 14^ 30–60% in coronary artery diseases,^15^ 26–69% in type 2 diabetes,^16, 17^ 24–37% in blood pressure,^18^ 40–70% in obesity^19^ and 58–66% in serum lipids level.^20^ Moreover, twin studies have revealed relatively modest genetic co-heritabilities (genetic correlations) between mood disorders and the different cardiometabolic abnormalities suggesting the influence of pleiotropic genes and shared biological pathways among them. For instance, the genetic correlation of depression with hypertension is estimated to be 19%, and between depression and heart disease is about 42%.^21^ The genetic correlation of depressive symptoms with plasma lipids level ranges from 10 to 31%,^22^ and 12% of the genetic component for depression is shared with obesity.^23^ Furthermore, gene–environment interactions can contribute to the cardiometabolic and mood disorders link. The interactions of genetic factors with stress, physical exercise, diet and lifestyle can influence the progression and pathogenesis of both cardiometabolic and mood disorders (Figure 1).^24, 25, 26^ These environmental factors might for example, modulate the expression of genes involved in the cardiometabolic pathways and a variety of pathways in the brain. Although it is at infancy stage, the ‘microbiome’ era has also revealed a range of complex interactions between environmental factors, genes and psychiatric disorders.^27^

In the last decade, substantial amounts of univariate (single disease) meta-analyses of genome-wide association studies (meta-GWASs) and candidate gene studies have been published. Indeed, the meta-GWASs and candidate gene studies have successfully identified a considerable list of candidate genes for major depressive disorder,^28^ bipolar disorder,^29^ coronary artery diseases,^30^ type 2 diabetes,^31^ hypertension,^26^ obesity,^32^ plasma lipids level,^33^ insulin and glucose traits^31, 34^ and blood pressure.^26, 35^

Despite the potential significance of studying pleiotropic genes and shared biological pathways, previous meta-GWAS and candidate gene studies were entirely focused on a single phenotype approach (single disease). A recent analysis of single-nucleotide polymorphisms (SNPs) and genes from the NHGRI GWAS catalog^36^ has showed as 16.9% of the genes and 4.6% of the SNPs have pleiotropic effects on complex diseases.^37^ Considering such evidence, we hypothesized that common genetic signatures and biological pathways mediate the mood disorders to cardiometabolic diseases relationship. In addition, these genes and their signalling pathways can influence the response to treatments in mood disorder patients (Figure 1). In this review, we systematically investigated the cardiometabolic diseases risk (CMD-R) genes that are possibly associated with mood disorders susceptibility, and with treatment response to MDD and BPD. We performed pathway and gene network analysis to provide additional insights in to the common pathways and biological mechanisms regulating mood disorders and the CMD-Rs. Understanding of these common pathways may provide new insights and novel ways for the diagnosis and treatment of comorbid cardiometabolic and mood disorders.

## Materials and methods

### Search strategy

#### Step 1: Identification of candidate genes for cardiometabolic diseases

We carried out a systematic search of candidate genes for the cardiometabolic diseases and/or associated risk factors. The National Human Genome Research Institute (NHGRI) GWAS catalogue,^36^ Westra *et al.*^38^ and Multiple Tissue Human Expression Resource (MuTHER)^39^ databases were used to identify the CMD-R genes. We reviewed meta-GWA study papers published until January 2016 for the diseases ‘type 2 diabetes’ or ‘coronary artery disease’ or ‘hypertension’ and (or) for the risk factors ‘blood pressure’ or ‘obesity or body mass index (BMI)’ or "plasma lipid levels (high-density lipoprotein, low-density lipoprotein, triglycerides, total cholesterol)’ or ‘insulin and glucose related traits (fasting glucose, fasting insulin, fasting proinsulin, insulin sensitivity, insulin resistance-HOMA-IR, beta cell function-HOMA-β and glycated haemoglobinA1C-HbA1C)’.

All GWAS significant SNPs (*P*<5 × 10^−8^) information (lead SNPs, reported genes, author(s), PubMed ID, date of publication, journal, discovery and replication sample sizes) was downloaded from the GWAS catalogue database. Additional information about the effect of the lead SNPs on nearby gene expression (*cis*-eQTLs) was collected from their respective publications. For the SNPs with no *cis*-eQTL information in their respective publications, we performed expression quantitative trait loci (*cis*-eQTL) analysis to verify the functional relationship between the reported genes and the lead SNPs using two publicly available databases: Westra *et al.*,^38^ and MuTHER.^39^ A CMD-R gene was considered as a candidate gene if, (1) at least one of the lead SNPs is located within or nearby to the gene; and (2) it is functionally relevant to influence at least one of the CMD-Rs as evidenced by gene expression analyses. We took the identified CMD-R genes forward for the second step literature review, as described below.

#### Step 2: Exploration of the role of cardiometabolic genes in mood disorders

In the second systematic review, we conducted a literature search in PubMed (MEDLINE database) for any genome wide association, candidate gene, or gene expression analysis study published in the fields of mood disorders and pharmacogenetics of mood disorders until January 2016. This step of the literature search was performed using SNIPPER tool (see web resources and tools). We considered studies that reported at least one of the CMD-R genes in ‘depression’ or ‘depressive disorder’ or ‘depressive symptoms’ or ‘MDD’ or ‘bipolar disorder’ or ‘mood disorder’ or ‘lithium treatment response’ or ‘Selective Serotonin Reuptake Inhibitors (SSRIs) treatment response’. A prior literature search implemented before the final review found that the majority of the genetic studies on treatment response to antidepressants and mood stabilizers were on lithium and SSRIs. As a result, the literature search on pharmacogenomics of mood disorders was limited to these predominant treatments.

### Inclusion criteria

General inclusion criteria of genetic studies that involve individuals of all ages in both sexes was implemented. The pharmacogenomics studies were restricted to only lithium or SSRIs treatment response in mood disorders.

### Exclusion criteria

Pharmacogenomics studies that used SSRIs or lithium for the treatment of psychosis, anxiety disorders, obsessive-compulsive disorder, post-traumatic stress disorder were excluded. We also excluded genetic studies that investigated drug-induced side effects of mood disorders.

## Biological pathway and network analysis

The potential pleiotropic genes were further explored to identify the most enriched canonical pathways and visualize gene networks using QIAGEN's Ingenuity Pathway Analysis (IPA, QIAGEN Redwood City, CA, USA, www.qiagen.com/ingenuity). For the analysis, all the 24 potential pleiotropic genes were entered as input into the software. IPA compares the proportion of input genes mapping to a biological pathway to the reference genes in the ingenuity databases. The significance of the overrepresented canonical pathways were determined using the right-tailed Fisher’s exact test later adjusted for multiple testing using the Benjamini-Hochberg (BH) method.^40^ Significance levels were determined at BH adjusted *P*-value <0.01. A gene network that connects the input genes with MDD, BPD and the cardiometabolic disorders was also generated.

### Web resources and tools

GWAS Catalogue: https://www.ebi.ac.uk/gwas/home

Westra *et al.* blood eQTL browser: http://genenetwork.nl/bloodeqtlbrowser/

MuTHER eQTL resource: http://www.muther.ac.uk/

SNIPPER tool v1.2: http://csg.sph.umich.edu/boehnke/snipper/

QIAGEN's Ingenuity Pathway Analysis: www.qiagen.com/ingenuity

## Results

### Characteristics of meta-GWA studies for the cardiometabolic disorders

**Figure 2:**
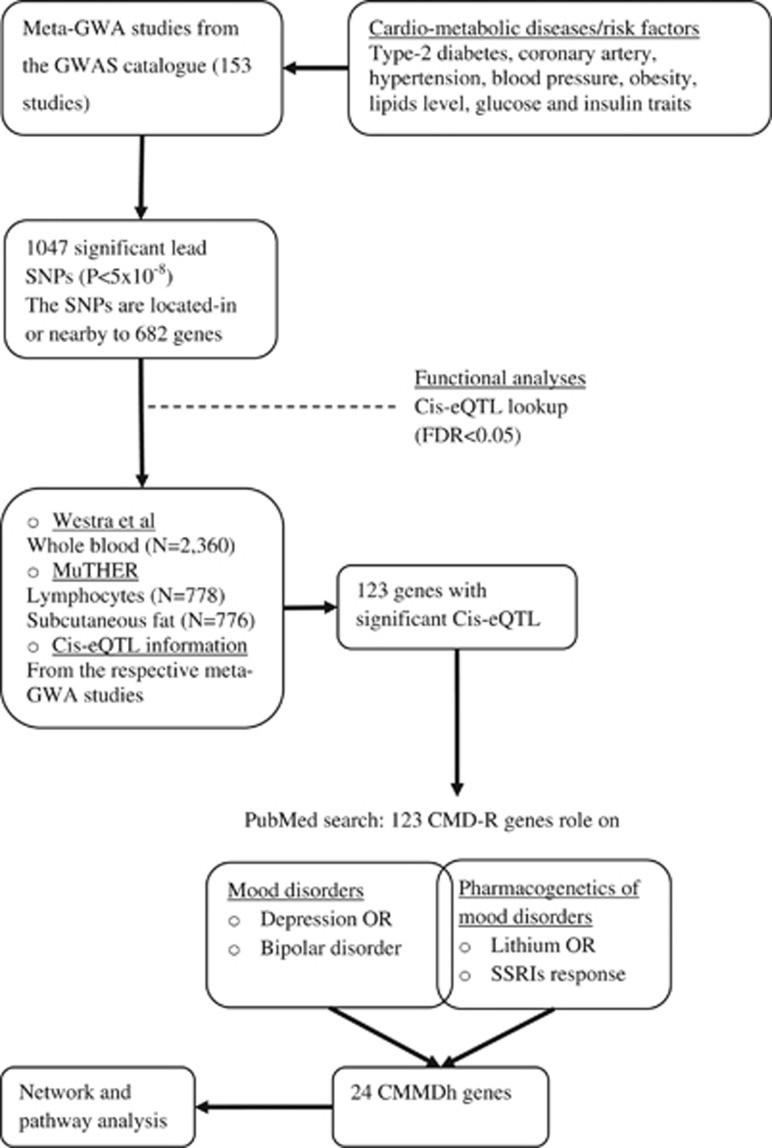
The flow chart shows the stages of literature search and evaluation of candidate pleiotropic genes for the CMD-Rs and mood disorders. CMD-R genes refers to the genes in which the CMD-R lead SNPs are located-in or nearby and their expression is influenced by the respective lead SNPs (*cis*-eQTL). CMD-R, Cardiometabolic Diseases and associated Risk factors; CMMDh, Cardiometabolic Mood Disorders hub genes; *cis*-eQTL, Cis (nearby) gene expression quantitative trait loci; GWAS, Genome Wide Assocation Study; Meta-GWA, meta-analysis of Genome Wide Association studies; MuTHER, Multiple Tissue Human Expression Resource; SNP, single nucleotide polymorphism.

The literature searches in the GWAS catalogue yielded 153 meta-GWA studies for the CMD-Rs: 38 studies for type 2 diabetes, 17 studies for coronary artery disease, 15 studies for hypertension and blood pressure, 26 studies for obesity and BMI, 37 studies for lipids and 20 studies for glucose and insulin traits (Figure 2). As shown in Figure 2, the meta-GWA studies reported 1047 lead SNPs and 682 nearby genes. Of these, 123 genes were functionally relevant to the cardiometabolic diseases and associated risk factors, as confirmed by gene expression analysis (*cis*-eQTLs). These genes were reviewed for their association with mood disorders and pharmacogenetics of mood disorders. Twenty-four of the 123 CMD-R genes have been implicated in mood disorders; and we named these genes the Cardiometabolic Mood disorders hub (CMMDh) genes.

**Figure 3:**
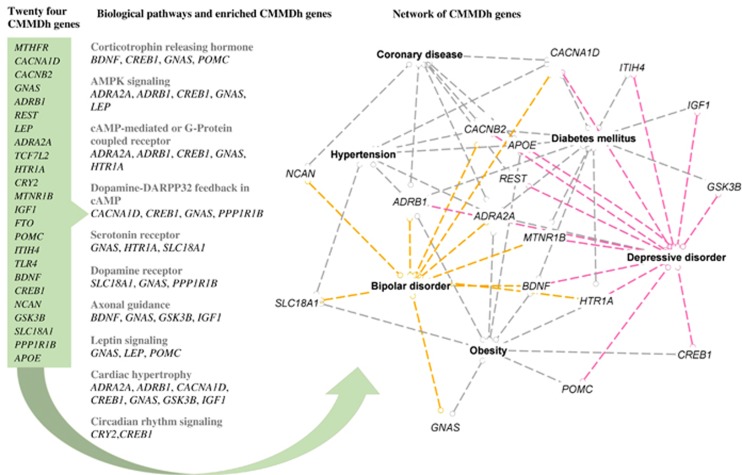
The list of 24 CMMDh genes (left), genes enriched to the top canonical signaling pathways (middle) and the network of these genes with mood disorders and the CMD-Rs (right). In the right, it illustrates ingenuity IPA-generated network of the CMMDh genes with coronary artery diseases, hypertension, diabetes mellitus, obesity, depressive disorder and bipolar disorder. The coloured dotted lines highlights CMMDh genes that were related to bipolar disorder (orange) and depression (red). CMMDh, Cardiometabolic Mood Disorders hub genes; IPA, Ingenuity Pathway Analysis.

Table 1 summarizes the 24 CMMDh genes and specific genetic variants across mood disorders and cardiometabolic diseases. These genes are *MTHFR*, *CACNA1D*, *CACNB2*, *GNAS*, *ADRB1*, *NCAN*, *REST*, *FTO*, *POMC*, *BDNF*, *CREB*, *ITIH4*, *LEP*, *GSK3B*, *SLC18A1*, *TLR4*, *PPP1R1B*, *APOE*, *CRY2*, *HTR1A*, *ADRA2 A*, *TCF7L2*, *MTNR1B*, and *IGF1* (for further details see Table 1). These genes were over-represented in the following biological pathways: corticotrophin-releasing hormone signaling *BDNF*, *CREB1*, *GNAS*, *POMC*; AMPK signaling *ADRA2A*, *ADRB1*, *CREB1*, *GNAS*, *LEP*; cAMP-mediated and G-protein coupled receptor signaling *ADRA2A*, *ADRB1*, *CREB1*, *GNAS*, *HTR1 A*; axonal guidance signaling *BDNF*, *GNAS*, *GSK3B*, *IGF1*; serotonin and dopamine receptors signaling *GNAS*, *HTR1A*, *SLC18A1, PPP1R1B*; dopamine-DARPP32 feedback in cAMP *PPP1R1B*, *CACNA1D*, *CREB1*, *GNAS*; leptin signaling *GNAS*, *LEP*, *POMC*; and the circadian rhythm signaling *CRY2*, *CREB1* (Table 2 and Figure 3).

We also performed a gene network analysis of the CMMDh genes to the mood disorders and cardiometabolic diseases. On the basis of the network analysis, the CMMDh genes were centrally involved in the link between mood disorders and the cardiometabolic diseases. For instance, *ADRB1* and *ADRA2A* genes linked the four most common cardiometabolic disorders (coronary diseases, hypertension, diabetes, obesity) with BPD and depressive disorder. The *CACNB2* and *CACNA1D* genes have shown network with coronary diseases, hypertension, diabetes, BPD and depression. Similarly, the other CMMDh genes acted as a hub between at least one of the cardiometabolic disorders and BPD and/or depression (Figure 3).

## Discussion

This, to the best of our knowledge, first cross-disorder review systematically evaluated candidate pleiotropic genes and biological pathways that are likely to be shared with mood disorders, cardiovascular diseases and metabolic disorders. We revealed 24 cardiovascular and metabolic disease genes implicated in depression, bipolar disorder or both. These genes belong to interrelated signaling pathways important in the hypotheses of both cardiometabolic diseases and mood disorders: corticotrophin-releasing hormone signaling, AMPK signaling, cAMP-mediated and G-protein-coupled receptor signaling, axonal guidance signaling, serotonin and dopamine receptors signaling, dopamine-DARPP32 feedback in cAMP signaling, leptin signaling and circadian rhythm signaling.

The corticotrophin-releasing hormone (CRH) signaling is one of the top canonical pathways that may underlie the link between CMD-Rs and mood disorders. This pathway comprises of CRH, CRH receptors (CRHR1, CRHR2), and other CRH-related peptides. It is the principal regulator of the HPA axis. There are consistent findings in the literature that support the role of the HPA axis dysregulation in mediating the risk of mood disorders and cardiovascular outcome.^41^ Our analysis found enriched CMMDh genes in the CRH signaling pathways (*BDNF*, *CREB1*, *GNAS* and *POMC*). Genetic variants of the genes for *BDNF*, *CREB1*, *GNAS* and *POMC* are associated with MDD,^42, 43^ BPD,^44^ obesity,^32, 45^ blood pressure and hypertension.^35, 46^ The genes belong to the group of stress responsive genes, and their activity could be modulated through the activation of the HPA-axis. In animal studies, the expression of *BDNF*^47^ and *CREB1*^[ref. 48]^ genes were dysregulated by chronic stress. It is therefore possible that an interaction of *BDNF*, *CREB1*, *GNAS*, and *POMC* genes with exposure to chronic stress or traumatic life events increase the risk of cardiometabolic and mood disorders either simultaneously, or through mediating factors. The CRH signaling pathway is the principal regulator of stress responses.^49^ Following an exposure to stress, the hypothalamus releases the CRH, stimulating the secretion of adrenocorticotrophic hormone from the anterior pituitary gland. This in turn stimulates the adrenal gland to produce glucocorticoids (principally cortisol). Cortisol will then act on several organs including the brain through its receptors.^49^ In acute conditions, the production of cortisol helps the body to fight pathogens (stress) and alleviate inflammation. However, when stressors are long lasting (chronic) they can cause cortisol receptor resistance and failure of the HPA-axis negative-feedback mechanism. This increases the duration and chronicity of inflammation, and a failure to downregulate the inflammatory response. Ultimately, failure in the HPA-axis processes may cause dysfunction in the brain and the body, causing both somatic diseases and brain disorders. Stress can either originate from the external environment as chronic extrinsic stress (CES) or within the internal body system as chronic intrinsic stress (CIS). Both CES and CIS can influence the CRH pathway genes mainly through gene expression and DNA methylation mechanisms.^50^

In relation to stress, there are two possibilities to explain mood disorders to cardiometabolic diseases association. The first is that the human body system may consider mood disorders or CMD-Rs as CIS and then dysregulate the HPA-axis through the CRH signaling pathways. Given that mood disorders tend to have an earlier age of onset compared to most of the CMD-Rs,^51^ they might be the primary CIS to induce cardiometabolic outcomes through the CRH signaling mechanism. Another possibility is that CES and/or CIS interact with the CRH signaling genes to cause both CMD-Rs and mood disorders. In either of the conditions, the CRH signaling genes interacts with the stressors to cause a dysfunction in the HPA-axis.

The second main canonical pathway was the adenosine monophosphate-activated protein kinase (AMPK) signaling pathway. This pathway regulates the intercellular energy balance. It inhibits or induces ATP consuming and generating pathways as needed. The pathway is especially important for nerve cells, as they need more energy with small energy reserves.^52^ Abnormalities in the pathway can disturb normal brain functioning. In animal studies, Zhu *et al.*, 2014 showed chronically stressed mice developed symptoms related to mood and metabolic abnormalities, such as significant weight gain, heightened anxiety, and depressive-like behavior. They also reported decreased levels of phosphorylated AMP-activated roteinkinase α (AMPKα), confirming the involvement of the AMPK pathway and its regulatory genes in metabolic disorders and depression.^53^ Recent studies also reported the activation of the AMPK pathway in rat hippocampus after ketamine treatment exerting rapid antidepressant effect.^54^ Major contributing CMMDh genes enriched in the AMPK pathway are *ADRA2A*, *ADRB1*, *LEP*, *CREB1* and *GNAS*. Variations in one or more of these genes can influence the activity of the AMPK pathway, subsequently impairing energy homeostasis in the brain and possibly in other cells.^52^ This could later cause energy shortages for the brain and somatic cells. Since brain cells are the most vulnerable units that require substantial amount of energy supply, any energy shortage would severely affect first the brain. Symptoms of mood change such as depressive behavior could emerge during this process. Moreover, AMP activation, for instance during stress, could induce insulin resistance promoting metabolic syndrome, that is, obesity, diabetes and cardiovascular diseases.^55, 56^ Hence, it is very likely that inappropriate activity of the AMPK pathway can imbalance the energy needs of the cells and be a cause to mood disorders and cardiometabolic diseases.

Axonal guidance signaling was also among the top overrepresented canonical pathways. The pathway is essentially related to neuronal connections formed by the extension of axons, which migrate to reach their synaptic targets. Axon guidance is an important step in neural development. It allows growing axons to stretch and reach the next target axon to form the complex neuronal networks in the brain and throughout the body. The patterns of connection between nerves depend on the regulated action of guidance cues and their neuronal receptors that are themselves encoded by axonal guidance coding genes. Activation of specific signaling pathways can promote attraction or repulsion and affect the rate of axon extension. One important observation in the axonal guidance pathway is the role of calcium and voltage-dependent calcium channels. The pathway is regulated by the entrance of calcium through the plasma membrane and release from intracellular calcium store. Calcium has been implicated in controlling axon outgrowth.^57^ CMMDh genes overrepresented in the axonal guidance-signaling pathway include the *BDNF*, *GNAS*, *GSK3B* and *IGF1* genes. Mutant axonal guidance genes followed by abnormal axon guidance and connectivity could cause a disorder primarily in the brain and subsequently to the peripheral organs.^58^

Other strong candidate mechanisms underlying mood disorders and cardiometabolic diseases are the serotonin and dopamine receptors signaling pathways. The serotonin pathway is mainly regulated by serotonin and its receptors known as 5-hydroxytryptamine receptors. Serotonin is a monoamine neurotransmitter synthesized in the central nervous system and its signaling modulates several physiological processes including regulation of appetite, mood and sleep, body temperature and metabolism. The *SLC18A1*, *HTR1A* and *GNAS* gene are among the CMMDh genes involved in the serotonin receptor-signaling pathway. The *SLC18A1* gene encodes for the vesicular monoamine transporter that transports for monoamines. Its function is essential to the activity of the monoaminergic systems that have been implicated in several human neuropsychiatric disorders.^59^ The *HTR1A* gene encodes a receptor for serotonin, and it belongs to the 5-hydroxytryptamine receptor subfamily. Dysregulation of serotonergic neurotransmission has been suggested to contribute for the pathogenesis of mood disorders^60, 61^ and it is implicated in the action of selective serotonin reuptake inhibitors.^62, 63, 64^ Animal studies have consistently demonstrated the influence of the serotonin pathway on both mood disorders and cardiometabolic disorders. Ohta *et al.*, 2011 have previously revealed as there is a converge in insulin and serotonin producing cells that can lead to metabolic diseases (diabetes) and mood disorders.^65^ The products of the insulin-producing cells (beta-islet cells) are involved to express the genes that synthesize serotonin, and serotonin also plays a role in the synthesis of insulin in the beta-islet cells.^65^

The dopamine receptors pathway, centrally regulated by dopamine, also appears to underlie the relationship between mood disorders and cardiometabolic diseases. Dopamine serves as a chemical messenger in the nervous system and its signaling has important roles in processes: emotion; positive reinforcement; motivation; movement; and in the periphery as a modulator of renal, cardiovascular and the endocrine systems.^66^ The *SLC18A1* and *GNAS* genes are among the CMMDh genes that belong to this pathway. The dopamine-signaling pathway further induces the dopamine-DARPP32 Feedback in cAMP signaling. The central regulator of this pathway is the *PPP1R1B* gene that encodes a bifunctional signal transduction molecule called the dopamine and cAMP-regulated neuronal phosphoprotein (DARPP-32). Other CMMDh genes in the pathway include *CACNA1D*, *CREB1*, and *GNAS*. The *CACNA1D* gene encodes the alpha-1D subunit of the calcium channels that mediates the entry of calcium ions into excitable cells. Calcium channel proteins are involved in a variety of calcium-dependent processes, including hormone or neurotransmitter release, and gene expression.^67^

Overall, genes that encode for molecules involved in HPA-axis activity, circadian rhythm, inflammation, neurotransmission, metabolism and energy balance were found to have a central role to link mood disorders with cardiometabolic diseases. It is also worth noting the gene–environment interaction that might contribute to the diseases.

## Implications of the review findings

Knowledge of genes and molecular pathways that are shared between mood disorders and cardiometabolic disorders have several important implications for future research and clinical practice. It is expected that increasing sample size, and consequently increasing power, will identify many more of the genes in the near future. Here we identify four implications of our findings.

First, the identification of shared molecular pathways implicated in disease susceptibility supports a growing evidence base for cross-diagnostic treatment paradigms. Shared molecular pathways could help to explain recent findings of reduced cardiovascular mortality,^68^ or improved diabetic control,^69^ in MDD patients treated with SSRIs. Second, further exploration of overlapping molecular pathophysiology has the potential to unveil novel targets for drug development, and may give clues for the re-purposing of existing medications.

Third, cardiometabolic disorders are associated with an increased risk of poor response to standard treatments in mood disorders.^70, 71^ Genetic profiling for cardiometabolic risk and stratified diagnosis of patients may help to classify treatment responders and treat them accordingly, thereby reducing the costs of ineffective exposure to medicines for the individuals and for the society. Early identification of at-risk individuals would also guide practitioner’s treatment recommendations, which may involve alternative somatic (for example, electroconvulsive therapy, repetitive Transcranial Magnetic Stimulation, ketamine) or specific psychological therapies as first- or second line treatments.

Fourth, studying the mechanisms of pleiotropic genes and shared pathways of mood disorders and somatic diseases could help untangle the clinical and genetic heterogeneity that characterizes these illnesses. It is possible that a ‘cardiometabolic’ endophenotype exists among mood disorders patients that may be identifiable through genetic profiling using polygenic scores or analysis of blood protein biomarkers. Preliminary evidence for such a phenotype, approximating the concept of ‘atypical depression’ characterized by increased appetite, weight gain and increased need for sleep, is emerging.^72, 73^ Working towards personalized care that allows for precise diagnostic, treatment and prevention strategies, research could then focus on genetically stratified patient cohorts instead of the very diverse patient pool currently diagnosed with MDD or BPD. There is a growing consensus that such stratification approaches have the potential to substantially improve the quality of mental health research and mental healthcare over the coming decades.^74^

Our review has limitations. Perhaps the most fundamental limitation was that almost all of the reviewed studies were performed in a univariate manner (single diseases approach). Essentially, multivariate models such as principal component analyses, multivariate mixed models and multivariate regression analyses are regarded as statistically powerful to perform cross-disorder analyses and identify pleiotropic genes. Unlike the multivariate approach, a univariate analysis investigates the association between a genetic variant and a single phenotype, aimed to identify genetic variants for individual diseases. Second, the review included studies that reported positively associated genes, and neither negative findings nor inconsistent evidences were assessed. We also found limited replication in some of the candidate genes, thereby demonstrating the necessity of future confirmatory studies. Third, only meta-GWAS were reviewed for the CMD-Rs and we implemented somewhat less stringent criteria for the genetic studies of mood disorders. GWAS for mood disorders have been less successful, mainly due to inadequate sample size and the phenotypic heterogeneity of the disorders. For this reason, the inclusion criteria for studies in these disorders was less strict. Hence, our review should be viewed as complementary to future mood disorders to cardiometabolic diseases gene investigation, providing an initial thorough summary of potential pleiotropic genes. Further population or case–control studies are necessary to confirm our proposed findings.

## Conclusion

Our review revealed potential pleiotropic genes and biological pathways that are likely to be shared between mood disorders and cardiometabolic diseases. Although the review provides some insight into common mechanisms and the role of pleiotropic genes, in-depth understanding of how these genes (and possibly others) mediate the association between mood disorders and cardiometabolic diseases requires future comprehensive cross-disorder research in large-scale genetic studies. This will enable us to better understand why patients suffer from multiple diseases, and how multi-morbidities influence pharmacological treatment response to diseases.

## Figures and Tables

**Table 1:** An overview of the 24 CMMDh genes shared between mood disorders and the cardiometabolic diseases

*Pleiotropic genes*	*Function of the coded protein*	*Polymorphisms associated with*	
		*Cardiometabolic disorders (lead SNP)*	*Mood disorders (description)*
*MTHFR*	The encoded MTHFR enzyme catalyzes the conversion of 5,10-methylenetetrahydrofolate to 5-methyltetrahydrofolate, a co-substrate for homocysteine remethylation to methionine. Methionine is an essential protein with multiple function in the brain and body.	Blood pressure rs17367504-G/A^46^	The common *MTHFR* C677T was associated with depression,^76^ and BPD.^77^ *MTHFR* gene polymorphisms interaction with childhood trauma increases the risk for depression.^78^
*CACNA1D*	Mediates the entry of calcium ions into cells	Blood pressure and hypertension rs9810888-G/T^26^	Rare variants in the calcium channel genes (*CACNA1B*, *CACNA1C*, *CACNA1D*, *CACNG2*) contribute to BPD^79^ and may influence treatment response to lithium.^80^
*CACNB2*	Mediates the entry of calcium ions into cells	Blood pressure rs4373814-G/C^35^ rs12258967-G/C^46^ rs11014166-A/T^81^	*CACNB2* gene polymorphisms were implicated in MDD and BPD.^82^
*GNAS*	Control the activity of endocrine glands through adenylate cyclase enzyme	Blood pressure and hypertension rs6015450-G/A^35^	SNPs in the *GNAS* gene were associated with BPD (rs6064714, rs6026565, rs35113254)^44^ and may influence antidepressant treatment response.^83^
*ADRB1*	Mediates the effects of epinephrine and norepinephrine	Blood pressure rs2782980-T/C^46^	Gly389 polymorphism of the beta-1 adrenergic receptor might lead to better response to antidepressant treatment in patients with MDD.^84^
*REST*	Regulate neurogenesis	Coronary artery disease rs17087335-T/G^30^	Reduced expression of *REST* in MDD patients at depressive state,^85^ and alteration in the expression of the *REST* gene was revealed in the brain of women with MDD.^86^
*LEP*	An appetite-regulating hormone that acts through the leptin receptor, functions as part of a signaling pathway that inhibits food intake and regulate energy.	Type 2 diabetes rs791595-A/G ^87^	SNPs in the leptin gene, decreased leptin gene expression and leptin deficiency in serum were related to antidepressant resistance.^88^ A significant reduction of the mRNA expression was found in the brain of MDD and suicidal patients.^89^
*ADRA2A*	Regulate neurotransmitter release from sympathetic nerves and from adrenergic neurons in the central nervous system	Type 2 diabetes or fasting glucose rs10885122-G/T^31^	*ADRA2A* gene polymorphisms (ADRA2A-1291G-male, ADRB2 Arg-female) were associated with sex-specific MDD,^90^ predicted antidepressant treatment outcome in MDD,^91^ and modified the effect of antidepressants for better improvement.^92^ However, they increased suicidal ideation during antidepressant treatment.^93^ Treatment with lithium produced an over expression of the *ADRA2A* gene in rats brain.^94^
*TCF7L2*	Regulate blood glucose homeostasis	Type 2 diabetes rs7903146-T/C^95^ Fasting glucose, proinsulin, insulin levels, or insulin resistance rs7903146-T/C^34^ rs4506565-T/A^31, 34^	Genome-wide association study of BPD in European Americans identifies a new risk allele (rs12772424-A/T) within the *TCF7L2* gene.^96^
*HTR1A*	Receptor for serotonin	Fasting insulin or insulin resistance rs16891077-A/G^97^	Variants in the *HTR1A* gene (rs6295, rs878567) were related to MDD and BPD.^60, 61^ A significant decrease in *HTR1A* mRNA levels in the brain of patients with MDD and BPD was found.^98^ Other polymorphisms (5-HT1A-1019G, Gly272Asp) in this gene were associated with antidepressant treatment response in MDD^62, 63, 64^ and in BPD.^63^ Increased DNA methylation in the promoter region of the *HTR1A* gene was also observed in patients with BPD.^99^
*CRY2*	Regulates the circadian clock	Fasting glucose or insulin rs11605924-A/C^31, 34^	Polymorphisms in *CRY2* gene were significantly associated with MDD^100^ and BPD.^100, 101^
*MTNR1B*	Receptor for melatonin that participate in light-dependent functions in the retina and brain. May be involved in the neurobiological effects of melatonin	Type 2 diabetes or plasma glucose level rs3847554-C/T^34^ rs10830962-C/G^102^ rs2166706-T/C^103^ rs10830963-G/C^31^ rs1387153-T/C^104, 105^	Gałecka *et al.* 2011 reported the significance of the *MTNR1B* gene polymorphism (rs4753426) for recurrent MDD.^106^ Additional SNP on the *MTNR1B* gene (rs794837) increased mRNA level in MDD patients.^106^
*IGF1*	Involved in mediating body growth and development	Fasting insulin, fasting glucose, or glucose homeostasis rs35767-G/A,^31^ rs35747-G/A^34^	Elevated levels of IGF-I was associated with MDD and antidepressant treatment response.^107^ A long-term deficiency of IGF-1 in adult mice induced depressive behaviour.^108^ Polymorphisms in the *IGF1* gene increased BPD risk.^109^ An over-expression of *IGF1* gene of BPD patients who respond well for lithium treatment was also reported.^110^
*FTO*	Regulates energy homeostasis, contributes to the regulation of body size and body fat accumulation. Studies in mice and humans indicate its role in body mass index, obesity risk, and type 2 diabetes.	Obesity rs7185735-G/A^32, 111^ Type 2 diabetes rs9936385-C/T^95^ HDL or triglycerides rs1121980-A/G^33^	The *FTO* gene variant (rs9939609-A/T) was associated with depression.^112^ Other variants of the *FTO* gene were involved in the mechanism underlying the association between mood disorders and obesity.^113^
*POMC*	Maintain the body"s energy balance and control sodium in the body	Obesity (BMI) rs713586-C/T^45^ rs1561288-T/C^114^ rs10182181-G/A^111^	Genetic variants in this gene were involved in treatment response to SSRIs (escitalopram or mirtazapine) in MDD patients.^115^
*ITIH4*	Involved in inflammatory responses	Obesity (BMI) rs2535633-G/C^116^	Genetic variants located in the regions of *ITIH1*, *ITIH3*, *ITIH4* genes were associated with BPD,^29^ and suicidal attempt in BPD patients.^117^
*TLR4*	Pathogen recognition and activation of innate immunity	Obesity (BMI) rs1928295-T/C^32^	The mRNA levels of the *TLR3* and *TLR4* genes were increased in depressed suicidal patients.^118^ *TLR4* gene expression was related to severity of major depression.^119^
*BDNF*	Promotes the survival of nerve cells	Obesity (BMI) rs2030323-C/A ^32, 111^ rs925946-T/G ^120^ rs10767664-A/T^45^	The Val66Met polymorphism was associated with depressive disorder,^42^ BPD^121^ and suicidal behavior in depressed and BPD patients.^122, 123^ It was also associated with SSRIs (escitalopram) response in depressed patients.^124^ A significantly decreased expression of the *BDNF* gene was observed in the lymphocytes and platelets of depressed patients.^125^ Treatment responsive depressive patients have also shown a decreased mRNA levels of the *BDNF* gene.^126^
*CREB1*	Involved in different cellular processes including the synchronization of circadian rhythmicity and the differentiation of adipose cells	Obesity rs17203016-G/A^32^	SNPs within this gene were associated with MDD risk in women ^43^ and antidepressants treatment resistance in MDD patients.^127^ An interaction of *CREB1* gene variants with *BDNF* variants predicted response to paroxetine.^128^ The *CREB1* gene variants (rs6785, rs2709370) increased BPD susceptibility^129^ and other SNPs on *CREB1* were suggested for BPD and lithium response.^130^
*NCAN*	Modulation of cell adhesion and migration	Total cholesterol rs2304130-G/A^131^ LDL cholesterol rs16996148-G/T^132^ rs10401969-C/T^133^ Triglycerides rs17216525-T/C^133^ rs16996148-G/T^132^	A SNP (rs1064395) in *NCAN* gene was found to be a risk factor for BPD in the European population.^134^ This SNP might resulted in a structural change of the brain cortex folding.^135^
*GSK3B*	Energy balance, metabolism, neuronal cell development, and body pattern formation	HDL cholesterol rs6805251-T/C^33^	Higher *GSK3B* activity was observed in MDD patients with severe depressive episode.^136^ Polymorphisms of this gene (rs334555, rs119258668, rs11927974) were implicated in MDD.^137^ In addition, rare variants in *GSK3B* gene increased BPD risk.^138, 139^ The *GSK3B* is a target gene for several mood stabilizers including lithium.^140, 141^
*SLC18A1*	Accumulate and transport neurotransmitters	Triglycerides rs9644568-A/G^142^ rs79236614-G/C^143^ rs326-A/G^144^	Variations in the *SLC18A1* (rs988713, rs2279709, Thr136Ser) gene confer susceptibility to BPD.^145^
*PPP1R1B*	A target for dopamine	HDL cholesterol rs11869286-G/C^33^	*DARPP-32* decreased in the prefrontal cortex of BPD patients,^146^ increased expression was also shown in BPD.^147^
*APOE*	Apolipoprotein E combines with fats (lipids) to form the lipoproteins. Lipoproteins are responsible for packaging cholesterol and other fats and carrying them through the bloodstream. APOE is the principal cholesterol carrier in the brain. There are at least three slightly different versions (alleles) of the APOE gene (E2, E3, and E4), of which E3 is the most common.	HDL, LDL or total cholesterol rs4420638-A/G^33^ rs1160985-C/T^148^ rs519113-C/G^149^	Genetic variation at the *APOE* gene contributed to depressive symptoms.^150^

Abbreviations: BPD, bipolar disorder; CMMDh, Cardiometabolic Mood Disorders hub genes; HDL, high-density lipoprotein; LDL, low-density lipoprotein; MDD, major depressive disorder; SNP, single nucleotide polymorphism.

**Table 2:** The top canonical signaling pathways enriched for the cardiometabolic mood disorders hub genes

*Canonical pathways*	*Enriched genes*	P*-value*^a^
Corticotrophin releasing hormone	*BDNF*, *CREB1*, *GNAS*, *POMC*	2.12 × 10^−5^
AMPK signaling	*ADRA2A*, *ADRB1*, *CREB1*, *GNAS*, *LEP*	9.24 × 10^−6^
cAMP-mediated	*ADRA2A*, *ADRB1*, *CREB1*, *GNAS*, *HTR1A*	1.71 × 10^−5^
G-Protein coupled receptor		2.18 × 10^−5^
Dopamine-DARPP32 feedback in cAMP	*CACNA1D*, *CREB1*, *GNAS*, *PPP1R1B*	5.28 × 10^−5^
Serotonin receptor	*GNAS*, *HTR1A*, *SLC18A1*	3.26 × 10^−5^
Dopamine receptor	*SLC18A1*, *GNAS*, *PPP1R1B*	1.21 × 10^−4^
Axonal guidance	*BDNF*, *GNAS*, *GSK3B*, *IGF1*	1.47 × 10^−3^
Leptin signaling	*GNAS*, *LEP*, *POMC*	1.17 × 10^−4^
Cardiac hypertrophy	*ADRA2A*, *ADRB1*, *CACNA1D*, *CREB1*, *GNAS*, *GSK3B*, *IGF1*	5.12 × 10^−8^
Circadian rhythm signaling	*CRY2*,*CREB1*	7.37 × 10^−4^

Abbreviations: AMPK, 5′ adenosine monophosphate-activated protein kinase; cAMP, cyclic adenosine 3′,5′-monophosphate; CMMDh, cardiometabolic mood disorders hub genes.

The table shows the top canonical pathways and enriched CMMDh genes as determined at BH adjusted *P*-value <0.01. The *P*-value indicates the likelihood of finding gene enrichment of the given pathway by chance.

^a^*P*-values were adjusted by Benjamini & Hochberg (BH) method.^40^
